# Severe Hypokalemia and Metabolic Alkalosis Caused by Licorice Discovered During the Treatment of Intraoperative Hypoxemia

**DOI:** 10.7759/cureus.25432

**Published:** 2022-05-28

**Authors:** Taisuke Shibata, Hiromi Yoshinuma, Daisuke Sugiyama, Osamu Kobayashi

**Affiliations:** 1 Department of Anesthesiology, Kameda Medical Center, Kamogawa, JPN

**Keywords:** licorice, intraoperative hypoxemia, herbal drugs, metabolic alkalosis, hypokalemia related medical emergencies

## Abstract

One of the causes of preoperative hypokalemia is the prolonged use of herbal medicines, especially licorice. Licorice can induce pseudo-aldosteronism, hypertension, metabolic alkalosis, and hypokalemia. An 87-year-old woman with a history of knee osteoarthritis was scheduled to undergo a total knee arthroplasty (TKA) under spinal anesthesia. She had also been prescribed herbal medicine for osteoarthritis of the knee two years before the surgery. During the surgery, the pulse oximeter showed hypoxemia. After the surgery was completed, arterial blood sampling showed hypoxemia, hypokalemia with electrocardiography (ECG) abnormalities, and metabolic alkalosis. The symptoms improved after the discontinuation of herbal medicines and administering potassium chloride. It is necessary to suspect electrolyte abnormalities as one of the causes of hypoxemia, hypertension, or ECG abnormalities in patients prescribed herbal medicines. Therefore, it is also important to ensure that patients on such drugs have their blood potassium levels assessed frequently in the perioperative period.

## Introduction

Intraoperative hypokalemia can cause various conditions, including arrhythmias, and it is important to check for the presence of hypokalemia preoperatively. One of the causes of preoperative hypokalemia is the prolonged use of herbal medicines, especially licorice [1]. Glycyrrhetinic acid, the active metabolite of licorice, inhibits 11-beta-hydroxysteroid dehydrogenase enzyme type 2 (11-β-HSD2), leading to a cortisol-induced mineralocorticoid response and a tendency toward sodium elevation and potassium reduction [2]. Thus, licorice can induce pseudo-aldosteronism, hypertension, metabolic alkalosis, and hypokalemia. We report a case of prominent intraoperative hypokalemia, which may have been caused by preoperative licorice intake.

## Case presentation

**Figure 1:**
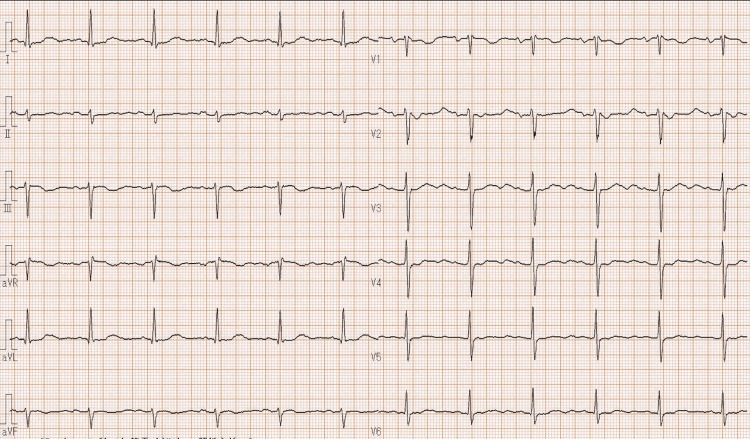
Electrocardiography three months before surgery. ECG shows normal sinus rhythm.

An 87-year-old woman with a body mass index of 25 kg/m^2^ and a history of knee end-stage osteoarthritis was scheduled to undergo a total knee arthroplasty (TKA) under spinal anesthesia. She was diagnosed with heart failure and mild lower leg edema. Blood tests the day before surgery showed a brain natriuretic peptide of 296.1 pg/mL, and chest radiography showed a cardiothoracic ratio of 67%. The patient's activities of daily living had been maintained, and she was able to exercise the equivalent of 4 METs, and her New York Heart Association functional classification was II. Her echocardiography showed normal wall motion, LVDd/Ds = 51/30 mm, ejection fraction = 73%, and moderate aortic regurgitation. She also had hypertension with a baseline of around 170/80 mmHg and was staged at the American Society of Anesthesiologists (ASA)-physical status classification III. Her medications included a loop diuretic (furosemide, 20 mg/day), calcium channel blocker (benidipine, 6 mg/day), angiotensin-II receptor blocker (telmisartan, 40 mg/day), and spironolactone (50 mg/day) for both heart failure and hypertension by oral intake. She had also prescribed the herbal medicines "syakuyakukanzoto (6 g/day)" and "kakkonto (7.5 g/day)" and a non-steroidal anti-inflammatory drug (celecoxib, 200 mg/day) with a proton-pump inhibitor (lansoprazole, 15 mg/day) for osteoarthritis of the knee two years before the surgery. Her prescription of herbal medicine was not known to us at the time of the anesthesiology preoperative consultation, and she continued to take the herbal medicine during the perioperative period. The day before surgery, her body temperature was 36.1 °C, her blood pressure was 160/80 mmHg, her heart rate was 77 bpm, and her oxygen saturation was 99% in room air. We did not measure her respiratory rate. Three months before surgery, her blood tests were unremarkable, including a K^+^ of 3.6 mEq/L, an Mg^2+^ of 0.82 mmol/L, and electrocardiography (ECG) also showed normal sinus rhythm (Figure 1). An echocardiogram showed an ejection fraction of 76% without any other abnormality on the day before surgery.

**Figure 2:**
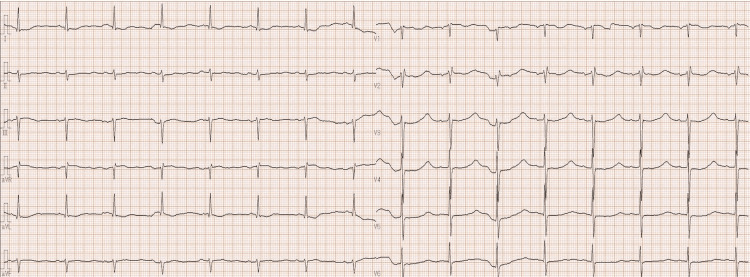
Electrocardiography just after the surgery. ECG shows QT prolongation, T-wave flattening, and prominent U-wave. ECG shows QT prolongation, T-wave flattening and prominent U-wave.

On the day of surgery, TKA was performed under spinal anesthesia as scheduled. On admission, the patient had an oxygen saturation in room air in the upper 80% range but promptly returned to 100% after a conversation and thus was given spinal anesthesia. We used 2 ml of 0.5% isobaric bupivacaine and punctured at the L3/4 level with an anesthesia level under Th 8 immediately after the surgery started and to below Th 5 by the end of the surgery. Because her oxygen saturation rose to 100% during a conversation, but it quickly dropped when she stopped speaking, 5 L/min of oxygen was initiated. A continuous infusion of dexmedetomidine (28 μg/h and a total amount of 40 μg) was started at the request of the surgeon. Atropine (0.5 mg) was also administered for a pulse rate below 40 bpm; however, there was no significant improvement. Although her blood pressure was 180/90 mmHg on admission, it fell to around 140/70 mmHg after the spinal anesthesia was administered. The surgery was completed after 2 hours and 17 minutes. A total of 450 ml of extracellular fluid was infused. The oxygen was stopped, and her SpO_2_ dropped to 80% in room air. We performed arterial blood sampling, which showed a K^+^ of 1.14 mEq/L and metabolic alkalosis (pH 7.508, paCO_2_ 66.3 mmHg, paO_2_ 75.1 mmHg, HCO_3_ 51.5 mmol/L, and BE 23.6 mEq/L). An ECG was obtained, showing QT prolongation, T-wave flattening, and a prominent U-wave (Figure 2). The patient was transferred back to the intensive care unit (ICU) for further management. In the ICU, the patient’s K^+^ improved to 2.8 mEq/L on postoperative day (POD) 1, and increased to 3.6 mEq/L on POD 2 and 4.2 mEq/L on POD 4 with the discontinuation of suspect drugs such as the loop diuretic, herbal medicines, and administering potassium chloride (KCl) both orally (5 times of the 13.4 mEq/g tablet) and intravenously (total 40 mEq). The ECGs obtained on PODs 1 and 2 showed a return to the normal range.

On POD 2, her paO_2_ was 55.0 mmHg with room air, her ECG normalized, and she was transferred to a general ward. Subsequently, her oxygenation gradually improved (paO_2_ 67.4 mmHg with room air), her K^+^ levels normalized, and she was discharged on POD 21 to home. Endocrine tests performed on transfer to the ICU revealed a low plasma renin activity of 0.1 ng/mL/h and a low plasma aldosterone level of 25.2 pg/mL. No thyroid hormone abnormalities were observed.

## Discussion

**Figure 3:**
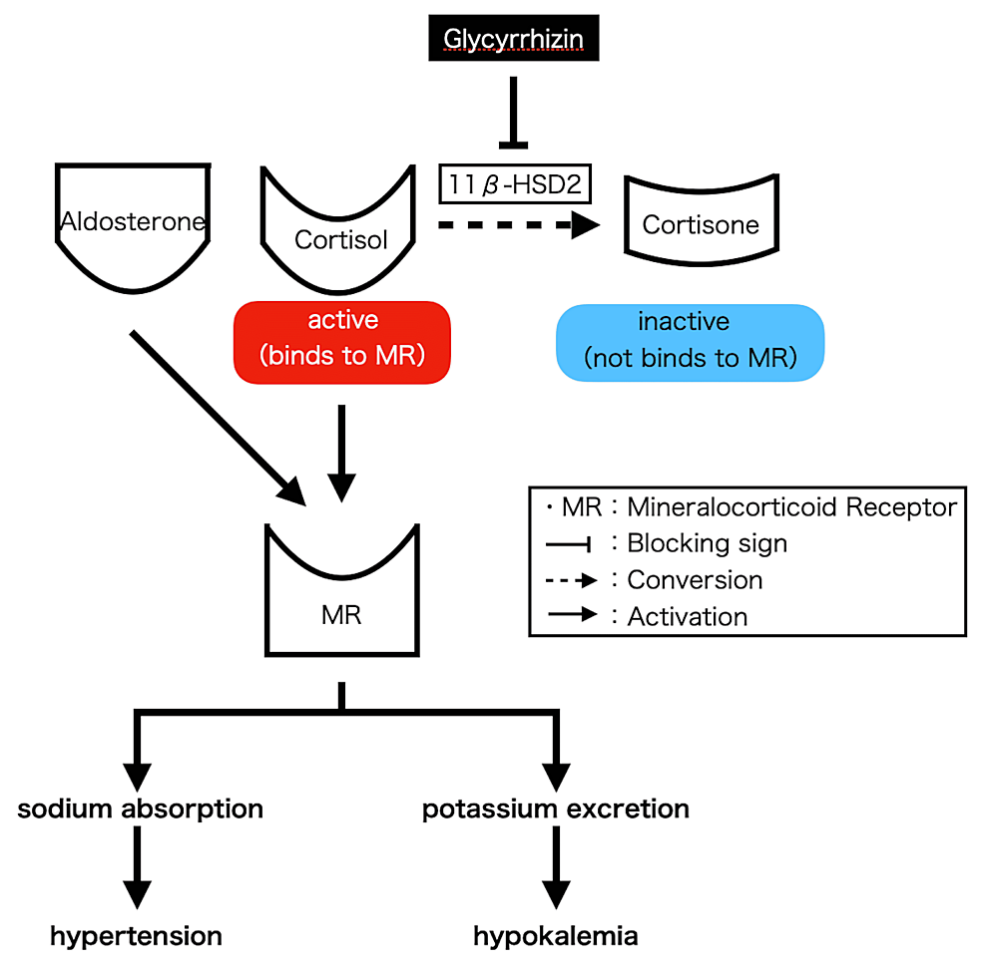
The mechanism that Glycyrrhizin can cause hypokalemia and hypertension. This figure is the authors' own creation.

This patient's possible cause of severe hypokalemia is pseudo-aldosteronism based on her history of taking herbal medicines that contained licorice because the hypokalemia was not observed during long-term treatment with loop diuretics or spironolactone, occurred during the period after she started taking the herbal medicine. The ASA has issued a handout on the perioperative use of herbal medicines and supplements [3]. Cortisol acts on mineralocorticoid receptors and promotes sodium absorption and potassium excretion. Cortisone, converted from cortisol by 11-β-HSD2, does not act on mineralocorticoid receptors. Glycyrrhizin, the main component of licorice, inhibits 11-β-HSD2. It results in a relative increase in cortisol, leading to excess sodium reabsorption and potassium excretion, which can cause hypertension, hypokalemia, metabolic alkalosis, low plasma renin activity, and low plasma aldosterone levels (Figure 3) [4-7]. This may be the reason the elevated blood pressure persisted after the induction of anesthesia. Generally, the most common cause of hypokalemia is diuretics [8]. A loop diuretic may be considered as the other cause of hypokalemia in this patient.

A possible cause of intraoperative hypoxemia is respiratory compensation of metabolic alkalosis. The patient was found to have severe alkalemia by arterial blood samples and may have been breathing less rapidly in an attempt to accumulate carbon dioxide (CO_2_) to compensate. She may have presented with hypoxemia as a result. Intraoperatively, the respiratory rate is one of the most critical indicators of metabolic acidosis and/or metabolic alkalosis. When the patient is managed on a ventilator, the respiratory rate is rarely a concern because the anesthesiologists set the respiratory rate, but it should be measured when hypoxemia and other symptoms are observed during conscious anesthesia. In addition, hypokalemia can cause muscle weakness and diaphragmatic dysfunction [9,10]. Although this patient had no subjective symptoms such as dyspnea, it is possible that the severe hypokalemia may have caused weakness of the respiratory muscles. Noninvasive diaphragmatic function assessment might have been considered preoperatively [11].

There are many licorice-based herbs in herbal medicines, and the herbal medicine this patient was taking provided an equivalent dose of 320 mg of glycyrrhizin per day for two years. It has been reported that taking 75 mg glycyrrhizin per day for two weeks could increase blood pressure, indicating that the risk of developing pseudo-aldosteronism was considerably higher in this patient [12]. Therefore, pseudo-aldosteronism was considered to have progressed between the time of examination three months before surgery and the day of surgery. Herbal medicine is one of Japanese most commonly used medicines, which makes it essential to evaluate serum K^+^ during the perioperative period.

## Conclusions

Herbal medicine that contains licorice is widely used in Japan and can lead to a drop in serum K^+^ and an increase in blood pressure. Because hypokalemia and high blood pressure can often be severe in the face of few subjective symptoms, it is necessary to suspect electrolyte abnormalities as one of the causes of hypoxemia, hypertension, or ECG abnormalities if they are observed. It is a common practice to discontinue all herbal medications and nutritional supplements seven days prior to surgery.
